# Comparison of Angiogenic, Cytoprotective, and Immunosuppressive Properties of Human Amnion- and Chorion-Derived Mesenchymal Stem Cells

**DOI:** 10.1371/journal.pone.0088319

**Published:** 2014-02-14

**Authors:** Kenichi Yamahara, Kazuhiko Harada, Makiko Ohshima, Shin Ishikane, Shunsuke Ohnishi, Hidetoshi Tsuda, Kentaro Otani, Akihiko Taguchi, Toshihiro Soma, Hiroyasu Ogawa, Shinji Katsuragi, Jun Yoshimatsu, Mariko Harada-Shiba, Kenji Kangawa, Tomoaki Ikeda

**Affiliations:** 1 Department of Regenerative Medicine and Tissue Engineering, National Cerebral and Cardiovascular Center Research Institute, Suita, Osaka, Japan; 2 Department of Biochemistry, National Cerebral and Cardiovascular Center Research Institute, Suita, Osaka, Japan; 3 Department of Gastroenterology and Hepatology, Hokkaido University Graduate School of Medicine, Sapporo, Hokkaido, Japan; 4 Department of Regenerative Medicine Research, Institute of Biomedical Research and Innovation, Kobe, Hyogo, Japan; 5 Division of Hematology, Department of Internal Medicine, Hyogo Medical College, Nishinomiya, Hyogo, Japan; 6 Department of Perinatology, National Cerebral and Cardiovascular Center, Suita, Osaka, Japan; 7 Department of Molecular Innovation in Lipidology, National Cerebral and Cardiovascular Center Research Institute, Suita, Osaka, Japan; 8 Department of Obstetrics and Gynecology, Mie University School of Medicine, Tsu, Mie, Japan; University of Minnesota Medical School, United States of America

## Abstract

Although mesenchymal stem cells (MSCs) can be obtained from the fetal membrane (FM), little information is available regarding biological differences in MSCs derived from different layers of the FM or their therapeutic potential. Isolated MSCs from both amnion and chorion layers of FM showed similar morphological appearance, multipotency, and cell-surface antigen expression. Conditioned media obtained from amnion- and chorion-derived MSCs inhibited cell death caused by serum starvation or hypoxia in endothelial cells and cardiomyocytes. Amnion and chorion MSCs secreted significant amounts of angiogenic factors including HGF, IGF-1, VEGF, and bFGF, although differences in the cellular expression profile of these soluble factors were observed. Transplantation of human amnion or chorion MSCs significantly increased blood flow and capillary density in a murine hindlimb ischemia model. In addition, compared to human chorion MSCs, human amnion MSCs markedly reduced T-lymphocyte proliferation with the enhanced secretion of PGE2, and improved the pathological situation of a mouse model of acute graft-versus-host disease. Our results highlight that human amnion- and chorion-derived MSCs, which showed differences in their soluble factor secretion and angiogenic/immuno-suppressive function, could be ideal cell sources for regenerative medicine.

## Introduction

Mesenchymal stem cells (MSCs) residing within various tissues, including bone marrow [1] and adipose tissue [2], are reported to differentiate into various types of cells including osteoblasts, chondrocytes, and adipocytes. This multipotency renders MSCs an attractive therapeutic source for regenerative medicine. However, because an invasive procedure is required to obtain autologous bone marrow or adipose tissue-derived MSCs, an alternative source of MSCs that can be obtained non-invasively is desirable.

Appendages of the fetus, which consist of the placenta, umbilical cord, and fetal membrane (FM), are normally discarded after delivery as medical waste. A large quantity of MSCs could be obtained without harm from the human FM because of its size (>40×40 cm), which represents an advantageous characteristic as a source of cell therapy. We have previously reported the therapeutic potential of rat FM-derived MSCs using various rat models including hindlimb ischemia, autoimmune myocarditis, glomerulonephritis, renal ischemia-reperfusion injury, and myocardial infarction [3]–[8]. Although the FM is composed of the amnion and chorion, and both layers contain MSCs [9], it is technically difficult to separate these membranes as well as their MSCs in rat.

Thus, the purposes of this study were: 1) to isolate and characterize MSCs from human amnion and chorion; 2) to examine their differences in the expression profile of growth factors and cytokines; and 3) to investigate the therapeutic potential and difference of these MSCs using murine hindlimb ischemia and acute graft-versus-host disease (GVHD) models.

## Materials and Methods

### Ethics Statement

The study protocol and informed consent procedure were approved by the ethics committee of the National Cerebral and Cardiovascular Center (Permit Number: M18-042-4). Animal protocols were approved by the Animal Care Committee of the National Cerebral and Cardiovascular Center Research Institute (Permit Number: 13052). Animal studies were conducted in strict accordance with the recommendations in the Guide for the Care and Use of Laboratory Animals of the National Institutes of Health. All animal surgery was performed under sodium pentobarbital anesthesia and all efforts were made to minimize suffering.

### Isolation and Expansion of Amnion- and Chorion-derived MSCs from Human FMs

After obtaining written informed consent, FMs were obtained following cesarean section of healthy donor mothers. Amnion and chorion were separated by mechanical peeling of the FM, and digested with type-II collagenase solution (5 ml/g tissue and 300 U collagenase/mL, Worthington Biochemicals, Lakewood, NJ) for 1 h at 37°C in a waterbath shaker. After filtration with a mesh filter, cells were suspended in α-minimal essential medium (α-MEM, Invitrogen, Carlsbad, CA) supplemented with 10% fetal calf serum (FCS, Hyclone, Logan, UT), 100 U/mL penicillin and 100 µg/mL streptomycin (Invitrogen), and incubated at 37°C with 5% CO_2_ after plating on a dish. The adherent, spindle-shaped MSCs developed visible symmetric colonies by days 1 to 2.

### Characterization of Human Amnion and Chorion MSCs

For defining FM-MSCs, we referred to the criteria proposed by the Mesenchymal and Tissue Stem Cell Committee of the International Society for Cellular Therapy [10].

Cultured MSCs were analyzed by FACSCalibur (BD Biosciences). Cells were incubated with fluorescein isothiocyanate (FITC) or phycoerythrin (PE)-conjugated monoclonal against human CD14 (clone M5E2), CD19 (clone HIB19), CD34 (clone 581), CD45 (clone HI30), CD73 (clone AD2), CD90 (clone 5E10), CD105 (clone 266), or HLA-DR (clone G46-6 (L243)), all purchased from BD Biosciences. Isotype identical antibodies served as controls.

To induce differentiation into osteocytes, MSCs were cultured in α-MEM with MSC osteogenesis supplements (Dainippon Sumitomo Pharma, Osaka, Japan) according to the manufacturer’s instructions. After 14–17 days of differentiation, cells were fixed and stained with Alizarin Red S (Sigma-Aldrich, St. Louis, MO).

To induce adipocyte differentiation, MSCs were cultured with adipocyte differentiation medium: 0.5 mM 3-isobutyl-1-methylxanthine (Wako Pure Chemical Industries, Osaka, Japan), 1 µM dexamethasone (Wako), 50 µM indomethacin (Wako), and 10 µg/mL insulin (Sigma-Aldrich) in α-MEM supplemented with 10% FCS. After 21 days of differentiation, adipocytes were stained with Oil Red O (Sigma-Aldrich).

### Conditioned Medium Analysis of FM-MSC-associated Cytoprotective Function

Human umbilical vascular endothelial cells (HUVECs; Lonza, Basel, Switzerland) were seeded onto a collagen-coated plate and incubated in medium 199 (Invitrogen) supplemented with 20% FCS for 24 h. Neonatal rat cardiomyocytes were isolated from Lewis rats on postnatal day 1, as described previously [11], and seeded onto a laminin-coated plate followed by incubation in α-MEM supplemented with 10% FCS for 24 h. Cells were then subjected to serum deprivation with/without hypoxia (1% O_2_) by culturing with serum-free medium or serum-free conditioned medium obtained from FM-MSCs cultured for 24 h. The cellular level of 3-(4,5-dimethylthiazol-2-yl)-5-(3-carboxymethoxyphenyl)-2-(4-sulfophenyl)-2H-tetrazo-lium (MTS), indicative of cell viability, as well as caspase-3 activity, was measured with a CellTiter96 AQueous One Solution Kit (Promega, Madison, WI) and a CaspACE™ Assay System Kit (Promega), according to the manufacturer’s instructions.

### Analysis of FM-MSC Production of Growth Factors and Prostaglandin E2

Conditioned media were collected from MSCs cultured in α-MEM with/without 10% FCS for 24 h (n = 4–6). The concentrations of the following growth factors were measured using ELISA kits: hepatocyte growth factor (HGF), insulin-like growth factor-1 (IGF-1), basic fibroblast growth factor (bFGF), vascular endothelial growth factor (VEGF), and prostaglandin E2 (PGE2), according to the manufacturer’s instructions (R&D Systems, Minneapolis, MN).

### FM-MSC Transplantation in the Hindlimb Ischemia Model

Six-week-old male KSN nude mice were anesthetized with pentobarbital, and the right common iliac artery was resected. After surgery, amnion MSCs (1×10^6^ cells/50 µL PBS), chorion MSCs (1×10^6^ cells/50 µL PBS), or PBS (50 µL PBS) was injected into the ischemic muscle with a 30-gauge needle at five different sites (n = 15 in each group). A laser Doppler perfusion image (LDPI) analyzer (Moor Instruments, Devon, UK) was used to measure serial hindlimb blood flow for 7 days, as previously described [12].

Five and seven days after MSC transplantation, ischemic hindlimb tissues were obtained and snap-frozen. Frozen tissue sections were stained with anti-mouse CD31 antibody (BD Biosciences) to detect capillary endothelial cells. Ten fields were randomly selected to count the number of capillaries. The adjusted capillary number per muscle fiber was used to compare the differences in capillary density between the three groups.

### In vitro CD4+ T cell Proliferation Assay

Peripheral blood mononuclear cells were prepared from buffy coats obtained from healthy donors by centrifugation through Ficoll-Paque (GE healthcare, Uppsala, Sweden). CD4+ T cells were isolated by magnetic bead depletion of CD8, CD14+, CD15+, CD16+, CD19+, CD36+, CD56+, CD123+, T cell receptor-gamma/delta, and glycophorin A–positive cells (CD4+ T Cell Isolation Kit) on an AutoMACS instrument (Miltenyi Biotec). CD4+ T cells (5×10^5^ cells/well) were cultured with X-VIVO medium (Lonza, Walkersville, MD) containing 2% FBS and 5 µg/ml anti-CD28 antibody (clone CD28.2, BioLegend, San Diego, CA) in anti-CD3-precoated 24-well culture plates (clone OKT3, BioLegend). During in vitro proliferation of CD4+ T cells, human amnion-, chorion-, or bone marrow-derived (Lonza) MSCs were co-cultured at 5×10^4^ cells/well. After 5 days of co-culturing, T cells were separated from the monolayer MSCs and counted with an automated cell counter (Countess, Invitrogen).

### FM-MSC Transplantation into the Acute GVHD Model

Seven- to eight-week-old female B6C3F1 (recipient; C57BL/6×C3H/He; H-2^b/k^) and BDF1 (donor; C57BL/6×DBA/2; H-2^b/d^) mice were purchased from Japan SLC (Shizuoka, Japan). Recipient mice were lethally irradiated with 15 Gy total body irradiation (TBI; X-ray) split into two doses separated by 2 h. On the following day, donor-derived cells (1×10^7^ bone marrow cells and 3×10^7^ spleen cells) were suspended in 0.2 mL RPMI-1640 medium (Invitrogen) and transplanted via the tail vein into the post-irradiation recipient mice. On days 14, 17, 21, and 25 after hematopoietic stem cell transplantation, 1×10^5^ amnion or chorion MSCs in 0.1 mL RPMI medium were transplanted via the tail vein. In the control group, the same amount of RPMI was infused via the tail vein. The severity of GVHD was evaluated by measuring the body weight of mice.

### Statistical Analysis

All values are expressed as mean ± standard error of the mean (S.E.M). Comparisons of parameters for more than three groups were made by one-way analysis of variance (ANOVA) followed by the Newman-Keuls’ test. Comparisons of the time-course of the LDPI index were made by two-way ANOVA for repeated measures, followed by Bonferroni tests. A p value <0.05 was considered statistically significant.

## Results

### Characterization of Amnion and Chorion MSCs

**Figure 1:**
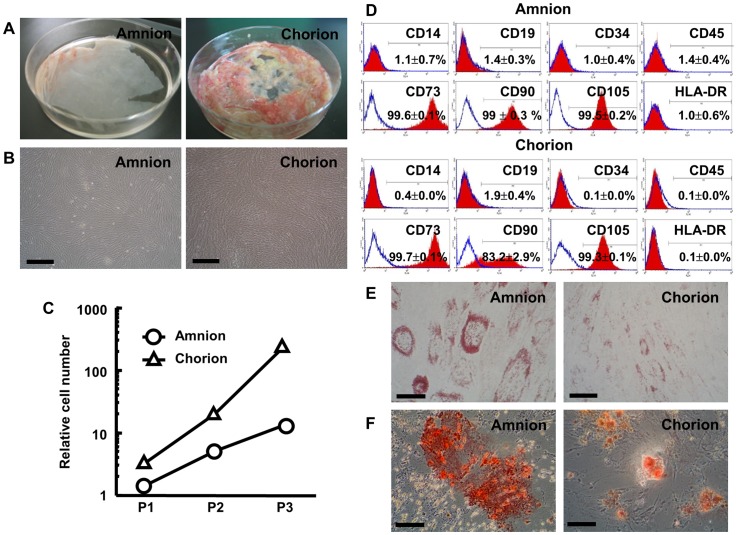
Characterization of human amnion- and chorion-derived MSCs. (A) Representative photographs of human amnion and chorion. (B) Photographs of cultured MSCs obtained from human amnion and chorion at passage 3. Scale bars = 500 µm. (C) Relative cell number of amnion- and chorion-derived MSCs at each passage. (D) FACS analysis of amnion and chorion MSCs. (E, F) Differentiation of amnion and chorion MSCs into adipocytes (E) and osteocytes (F). Scale bars = 100 (E) and 50 (F) µm.

From each human FM, 23.5±3.7 g amnion and 37.6±2.5 g chorion could be separated (n = 5 and n = 3, respectively) (Figure 1A). By enzymatic digestion, over one million cells per gram of the amnion (1.9±0.2×10^6^/g, n = 5) or chorion (1.3±0.3×10^7^/g, n = 3) were obtained. At passage 3, cultured cells from both layers were fibroblast-like, spindle-shaped cells, and there was no difference in morphology according to the origin of layers (Figure 1B). Cell-doubling time of amnion MSCs (32.2±1.13 h) was equal to that of chorion MSCs (34.1±1.94 h) (Figure 1C).

Both amnion- and chorion-derived MSCs expressed CD73, CD90, and CD105, but not CD14, CD19, CD34, CD45, or HLA-DR (Figure 1D), which satisfied the criteria for identifying MSCs [10]. In addition, amnion and chorion MSCs could differentiate into adipocytes and osteocytes, as demonstrated by positive Oil Red O and Alizarin Red S staining, respectively (Figure 1E and 1F).

### Cytoprotective Effects of Amnion and Chorion MSCs on Endothelial Cells and Cardiomyocytes

**Figure 2:**
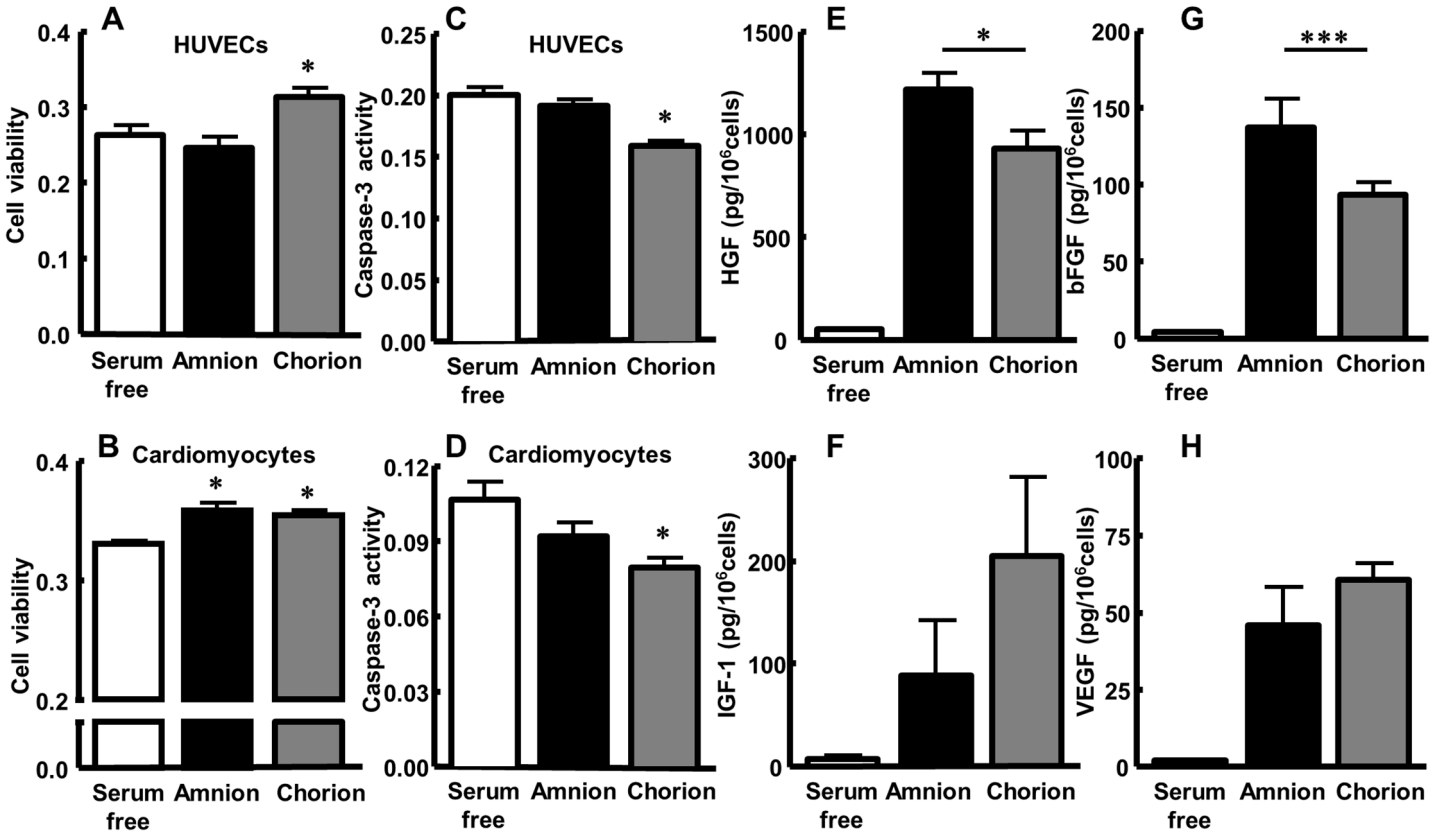
Growth factor secretion and the cytoprotective effect of amnion and chorion MSCs. (A–D) Cytoprotective effect of FM MSC-derived conditioned medium was analyzed by the MTS assay (A, B) and caspase-3 activity (C, D) in HUVECs (A, C) and cardiomyocytes (B, D). Values are mean ± SEM. *p<0.05 vs. serum-free. (E–H) Conditioned medium obtained from FM-derived MSCs was collected after incubation for 24 h. The concentration of HGF (E), IGF-1 (F), bFGF (G), and VEGF (H) in serum free conditioned medium was measured by ELISA. *p<0.05 and ***p<0.001.

To evaluate the cytoprotective effect of amnion and chorion MSCs, we examined cell viability and apoptosis of HUVECs and neonatal rat cardiomyocytes cultured under serum deprivation. In the MTS assay, cell viability of cardiomyocytes was significantly increased when cultured with conditioned medium obtained from amnion and chorion MSCs (absorbance value: serum-free control 0.331±0.002, amnion MSCs 0.359±0.006; p<0.001, and chorion MSCs 0.355±0.004; p<0.01 vs. control) (Figure 2B). Cell viability of HUVECs also increased when cultured with chorion MSC-derived conditioned medium (serum-free control 0.263±0.013, amnion MSCs 0.247±0.014, and chorion MSCs 0.313±0.012; p<0.05 vs. control) (Figure 2A). Similarly, conditioned medium obtained from chorion MSCs significantly decreased the caspase-3 activity of HUVECs (absorbance value: serum-free control 0.201±0.006 vs. chorion MSCs 0.159±0.004; p<0.001) and cardiomyocytes (control 0.106±0.007 vs. chorion MSCs 0.079±0.004; p<0.05) (Figure 2C, D). Amnion MSC-derived conditioned medium also showed a tendency to decrease the caspase-3 activity of these cells, but without statistical significance.

### Secretion of Growth Factors from Cultured Amnion- and Chorion-derived MSCs

To investigate the secretion of major growth factors from MSCs, we performed ELISA of HGF, IGF-1, bFGF, and VEGF. The differences in the cellular expression profile of the growth factors were observed in these FM-derived MSCs (Figure 2E–H). Among these growth factors, amnion MSCs secreted significant amounts of HGF (1217.2±80.2 pg/10^6^ cells; p<0.001 vs. chorion-MSC) and bFGF (137.2±18.5 pg/10^6^ cells; p<0.05 vs. chorion-MSC) compared with chorion MSCs (HGF: 932.5±85.3 pg/10^6^ cells, bFGF: 93.6±8.1 pg/10^6^ cells) (Figure 2E, G). There was no significant difference between amnion and chorion MSCs in the level of secreted IGF-1 (88.8±53.4 pg/10^6^ cells and 205±77.0 pg/10^6^ cells, respectively) and VEGF (46.1±12.3 pg/10^6^ cells and 60.7±5.3 pg/10^6^ cells, respectively) (Figure 2F, H).

### Augmentation of Angiogenesis in the Ischemic Hindlimb after Human FM-MSC Transplantation

**Figure 3:**
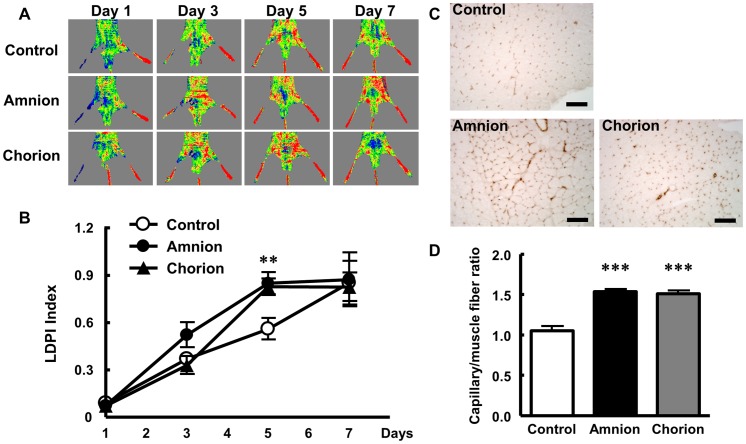
Angiogenic potential of amnion and chorion MSCs against hindlimb ischemia. (A) Representative images of serial hindlimb blood perfusion. Blood perfusion of ischemic hindlimb increased in the amnion and chorion MSC groups at day 5. (B) Quantitative analysis of hindlimb blood perfusion with the LDPI index, the ratio of ischemic to non-ischemic hindlimb blood perfusion. (C) Representative photographs of immunohistochemistry with anti-CD31 antibody. Scale bars = 100 µm. (D) Quantitative analysis of capillary density in ischemic hindlimb muscle at day 5 among the control, amnion, and chorion MSC groups. Capillary density is shown as the capillary-to-muscle-fiber ratio. Data are mean ± SEM. **p<0.01 and ***p<0.001 vs. control.

Analysis of LDPI revealed that accelerated limb perfusion was observed in the amnion and chorion MSC-transplanted groups (Figure 3A). The LDPI index was significantly higher in the amnion and chorion MSC groups (amnion MSCs: 0.85±0.07; p<0.01, chorion MSCs: 0.83±0.05; p<0.01) than in the control group (0.56±0.07) 5 days after transplantation (Figure 3B). At 7 days after transplantation, there was no difference between the treated and control groups.

Immunostaining with the endothelial marker CD31 showed significant augmentation of capillaries in the amnion and chorion MSC-treated groups compared with the control group (Figure 3E). The capillaries-to-muscle-fiber ratio of ischemic muscle at day 5 after transplantation was significantly increased in the amnion and chorion MSC groups (amnion MSCs: 1.53±0.03/muscle fiber; p<0.001, chorion MSCs: 1.51±0.04/muscle fiber; p<0.001) compared with the control group (1.05±0.06/muscle fiber; Figure 3F). At day7, the capillaries-to-muscle-fiber ratio of ischemic muscle was also increased in the amnion or chorion MSC-transplanted mice (amnion MSCs: 1.67±0.17/muscle fiber, chorion MSCs: 1.43±0.09/muscle fiber) compared to the control mice (1.36±0.11/muscle fiber).

### Immunosuppressive Property of Human FM-MSCs

**Figure 4:**
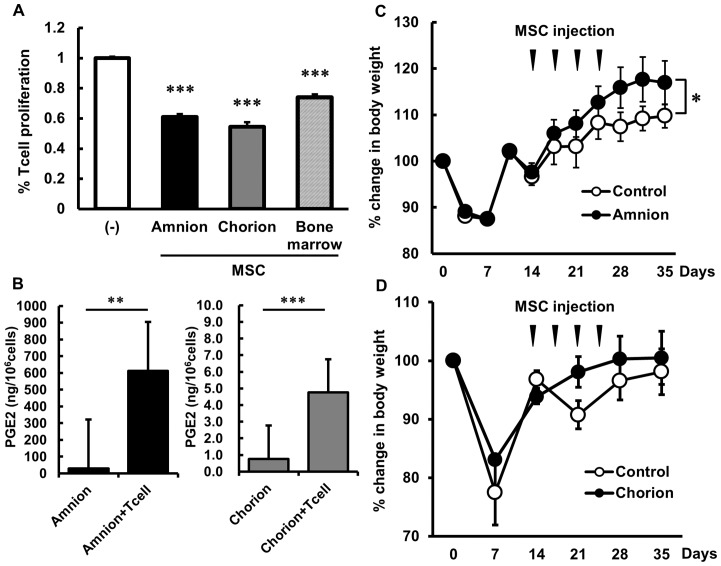
Immunosuppressive property of amnion and chorion MSCs. (A) Inhibition of human CD4+ T cell proliferation upon co-culture with human amnion, chorion, and bone marrow MSCs. (B) The concentration of PGE2 in FM-MSC-conditioned medium was measured by ELISA. Amnion MSCs secreted a significant amount of PGE2 compared with chorion MSCs. (C, D) Effect of human amnion (C) or chorion (D) MSC transplantation in a murine GVHD model. Treatment with amnion MSCs significantly reduced recipient weight loss in a mouse model of GVHD. *p<0.05, **p<0.01 and ***p<0.001.

Although the number of T cells was markedly increased under proliferating conditions of human CD4+ T cells stimulated with anti-CD3 and -CD28 antibodies, the increase was significantly suppressed when co-cultured with amnion-, chorion-, or bone marrow-derived MSCs (61.1±1.8%, 54.6±3.0%, 74.0±2.1%, respectively. p<0.001 vs. control) (Figure 4A).

PGE2 is a well-known immune modulator in bone marrow MSCs [13] and we confirmed that amnion MSCs in culture secreted a significant amount of PGE2 (29.7±7.8 ng/10^6^ cells), particularly when co-cultured with human CD4+ T cells (613.1±139.9 ng/10^6^ cells; p<0.01 vs. amnion MSCs) (Figure 4B). In chorion MSCs, however, the concentration of PGE2 was relatively low (0.77±0.13 ng/10^6^ cells) but significantly increased in co-culture with CD4+ T cells (4.76±0.47 ng/10^6^ cells; p<0.001 vs. chorion MSCs). The experiments were repeated with two or three independent MSC/CD4+ T cell donor pairs and the data are presented as the measured mean levels.

In addition, to evaluate the potential of FM-MSCs to suppress acute GVHD, mice underwent allogeneic hematopoietic stem cell transplantation and treatment with human FM-MSCs. As shown in Figure 4C, the loss in body weight of recipient mice after allogeneic hematopoietic stem cell transplantation was significantly reduced with concomitant transplantation of human amnion-derived MSCs. In human chorion MSC-transplanted group, however, no significant changes in body weight was observed during the observation period (Figure 4D).

## Discussion

Human MSCs derived from bone marrow or adipose tissue exert a regenerative effect in animal models and human patients [14]. In addition, several reports have described the therapeutic potential of transplanted cells derived from the appendages of the fetus, including amniotic epithelium cells [15], and amniotic fluid- [16], amnion-, and chorion-derived MSCs [17], [18]. We have previously demonstrated the therapeutic potential of rat FM-MSCs using various rat models including hindlimb ischemia, autoimmune myocarditis, glomerulonephritis, renal ischemia-reperfusion injury, and myocardial infarction [3]–[8]. Recent studies including ours also revealed the angiogenic and immunosuppressive property of human fetal appendage-derived MSCs [14], [18]–[20], but comparative studies of the therapeutic effects among these MSCs are lacking. Therefore, in this study, we examined the differences in the cellular function and therapeutic properties between human FM-derived amnion and chorion MSCs.

It is known that MSCs exert their regenerative effects through differentiation into specific cell types, but recent studies suggest that their ability to stimulate regenerative effects is mainly induced via paracrine effects [3], [4], [8], [21]. This theory is substantiated by several reports that MSCs secrete various growth factors and cytokines including VEGF, IGF-1, HGF, adrenomedullin (AM), and PGE2 [3]–[5], [8], [21], [22]. In this study, we first confirmed that chorion MSCs as well as amnion MSCs secreted significant amount of these soluble factors, which would contribute to accelerating regenerative effects. Compared with chorion MSCs, amnion MSCs secreted significantly larger amounts of HGF and bFGF. However, amnion MSCs secreted less IGF-1 compared to chorion MSCs. We assume that these differences in the cytokine expression profile might reflect the angiogenic and cytoprotective properties of amnion and chorion MSCs, as we observed difference in the effect on endothelial cells and cardiomyocytes in our conditioned-medium analysis. However, the actual function of amnion or chorion MSC-derived cytokines should be further investigated *in vivo* because both human amnion and chorion MSC transplantation similarly induced angiogenesis in the hindlimb ischemia model.

Previous reports have shown that PGE2 is a major modulator of the MSC-induced anti-inflammatory response [13]. In this study, a noteworthy finding was a distinctly high concentration of PGE2 in amnion MSCs in comparison with chorion MSCs, particularly when co-cultured with CD4+ T cells. Because of their high PGE2 production, human amnion MSCs might be a better cell source from an immunosuppressive point of view. In fact, we proved for the first time that human amnion MSCs, but not chorion MSCs, improved the pathological situation of an acute GVHD model. Because our previous study demonstrated that human amnion MSCs markedly inhibited differentiation as well as proliferation of Th1/Th17 cells [6], human amnion MSCs could effectively suppress Th1/Th17 immunity and improve outcome in GVHD.

The merit of using FMs lies in that they are free form ethical concern and that a large number of MSCs can be obtained considering the size of FM. As more than one or ten million MSCs per gram of the amnion or chorion could be obtained, more than 10^9^ or 10^10^ MSCs could theoretically be obtained at passage 3 within one month, respectively. Now we are planning to initiate clinical studies with human amnion MSCs in acute GVHD and Crohn’s disease, and we need more than 10^10^ MSCs for the treatment of one patient. We are convinced that human FM-MSCs are an attractive source for cell therapy because of their easy availability compared with other somatic, embryonic stem, and iPS cells.

In conclusion, both amnion and chorion MSCs have angiogenic, cytoprotective, and immunomodulatory effects. Because of high PGE2 production and immunosuppressive properties, human amnion MSCs have the advantage for the treatment of immune-related diseases. In addition, since a large number of MSCs could be obtained from FMs, human amnion and chorion MSCs would be a useful cell source for regenerative medicine.
